# Hysteroscopic Removal of an Unwanted Remainder in the Uterus after Two Years of Caesarean Section: A Case Report of Gossypiboma

**DOI:** 10.4314/ejhs.v34i2.9

**Published:** 2024-03

**Authors:** Faiza Ejaz, Zahid Hyder Wadani, Farheen Yousuf

**Affiliations:** 1 Department of Obstetrics and Gynaecology, Aga Khan University, Stadium Road, PO Box 3500, Karachi, Pakistan

**Keywords:** Gossypiboma, Retained Surgical Items, Uterus

## Abstract

**Background:**

Retained surgical items (RSIs) constitute a rare complication arising after surgical procedures. Their occurrence may be averted through diligent precautionary measures. Perioperative counting of equipment and materials is the most common method of screening for RSIs. Subsequent confirmation of the diagnosis can be achieved through clinical examination and imaging studies.

**Case:**

We report a case of successful hysteroscopic identification and retrieval of gauze inadvertently left within the uterine cavity, after a cesarean section two years back. The patient was later diagnosed with RSIs during routine evaluation for secondary subfertility and vaginal discharge.

**Conclusions:**

Despite the relatively low incidence of RSIs, they represent a significant and preventable source of patient harm, carrying the potential for fatal outcomes and resulting in substantial medical and legal expenditures.

## Introduction

The term “Gossypiboma” originates from the Latin word “Gossypium”, which means “cotton”, and the Swahili term “boma”, which denotes a “place of concealment”. This term is used to indicate the inadvertent retention of a cotton pad or surgical sponge within the peritoneal cavity following a surgical procedure (1). RSIs will remain a worldwide medical error concern as long as nonabsorbable materials continue to be utilized in surgical procedures. Despite the universal implementation of manual counting protocols for surgical textiles and instruments, incidents of RSIs continue to rise (2). Gauzes account for 90% of the RSIs due to their frequent use during surgical procedures (1, 2). While RSIs are primarily attributable to human error, unintentional RSIs contribute for 70% of cases which require reinterventions, resulting in an alarming morbidity rate of 80% and a mortality rate of 35% (2). RSIs are estimated to occur in 1 out of 10,000 surgeries, and the risk is increased by nine-fold in emergency surgeries (3). The aim of this case report is to raise awareness regarding RSIs as a potential differential diagnosis when a patient with prior surgical history presents with nonspecific symptoms.

## Case Presentation

A 29-year-old female, who had previously undergone a cesarean section in 2019, presented with a complaint of profuse foul-smelling vaginal discharge shortly after her cesarean section. The vaginal discharge eventually resolved; however, the patient experienced a two-year period of infertility following the 2019 procedure. During the evaluation of her symptoms, the patient denied any history of abdominal pain, bowel disturbances, urinary issues, menstrual irregularities, or signs of sepsis. Physical examination revealed stable vital signs, a soft and non-tender abdomen, and no evidence of visceromegaly upon deep palpation.

**Figure 1:**
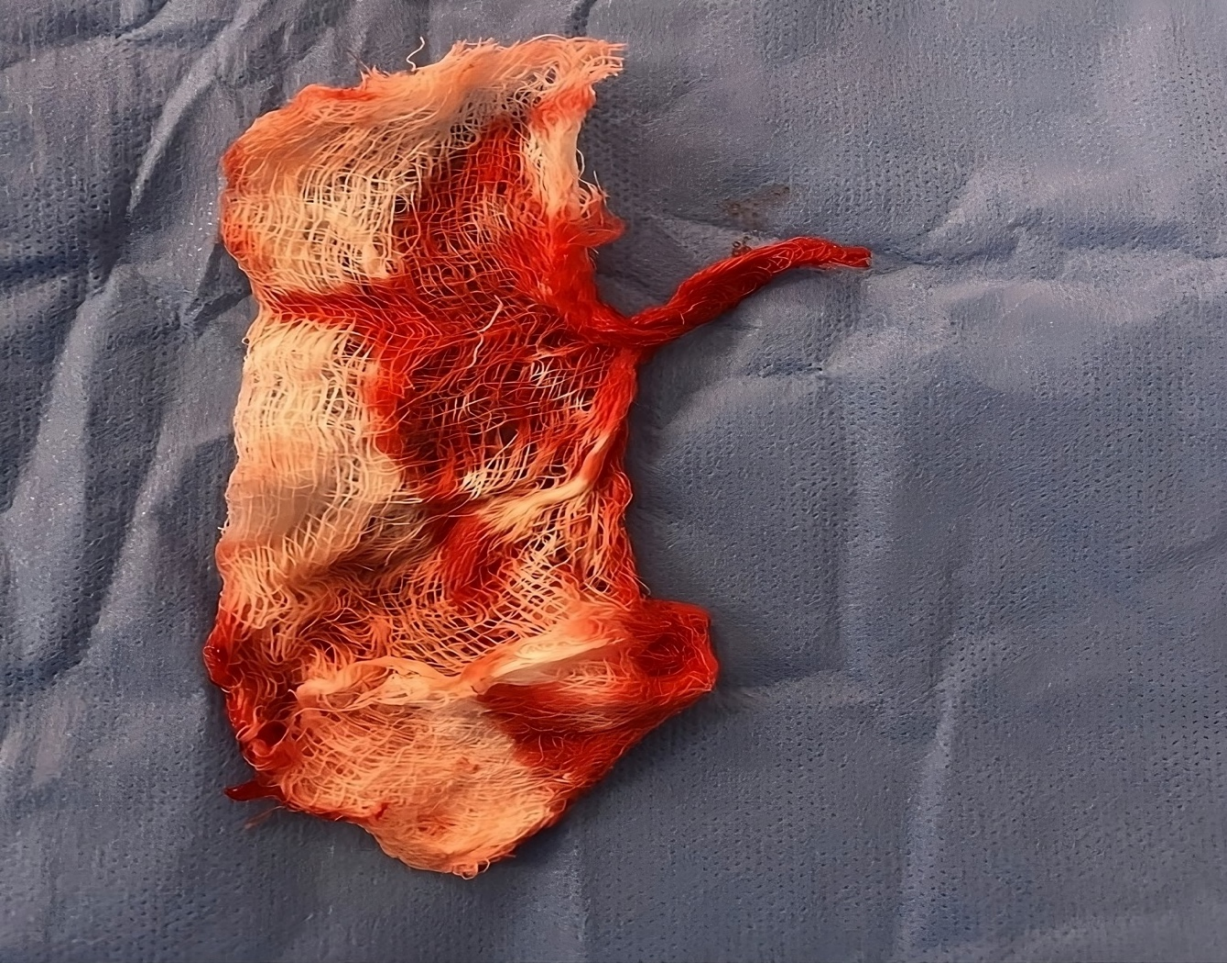
A gauze piece removed after hysteroscopic surgical procedure

In 2021, a transvaginal ultrasound revealed an endometrial thickness of 0.4 cm, with a dense echogenic structure measuring 5.2x1.8 cm within the uterine cavity, producing strong shadowing suggestive of a foreign body. No free fluid was observed in the pelvic region. An informed consent was obtained from the patient and diagnostic hysteroscopy was performed to remove the foreign body from the uterus. During the procedure, a gauze piece measuring 4x4 cm (Figure 1) was identified in the anterior fundal region of the uterus and removed, resulting in mild bleeding from the endometrial bed. The uterine size and endometrial appearance were normal upon inspection. A specimen was sent for histopathological analysis, which identified the presence of Escherichia Coli and Staphylococcus Aureus on culture. The patient received intravenous antibiotics, necessitating an extended post-operative hospital stay, during which she remained clinically stable. Subsequently, the patient successfully achieved spontaneous conception within six months and delivered a healthy baby.

## Discussion

Among the case reports on identification and removal of gossypiboma from the abdominal cavity, a minority have been identified during investigations aimed at elucidating the causes of subfertility (4).. In this report, we presented a similar kind of case of RSIs, which was identified while investigating reasons of subfertility.

The incidence of unintentional RSIs is likely underestimated, a phenomenon partially attributed to the challenges associated with making a diagnosis. Moreover, this underestimation may also stem from potential legal consequences and the complexities surrounding the reporting of medical malpractice and managing its repercussions (2). In most instances, the underlying causes of RSIs can be traced back to communication errors and poor team dynamics. Such cases have adverse implications for both healthcare providers and the reputation of the medical institution. Therefore, prioritizing prevention is of utmost importance. The rigorous and systematic counting of all surgical items stands as the most effective strategy. It is not uncommon to encounter discrepancies during the counting process, which are typically resolved through recounting. Nevertheless, it is essential to acknowledge that human error, imperfections, and excessive workloads can, at times, inevitably contribute to instances of RSIs.

The Association of periOperative Registered Nurses (AORN) recommended a comprehensive counting protocol at various critical junctures throughout surgical procedures. This protocol encompasses an initial count before the commencement of any surgical procedure, a count at the point of addition of a new item, a count preceding the closure of a cavity within a cavity, a count upon incision closure, and a final count at skin closure. In the event of any count discrepancy, it is the duty of the entire surgical team to collectively investigate and locate the missing items (3).

Imaging modalities are used in the diagnosis of RSIs. The initial choice is plain radiography, although it carries a notable false-negative rate ranging from 10% to 25%. Computed tomography serves as the secondary imaging technique for the purpose of ruling out the presence of gossypiboma (5). Moreover, recent technological advancements, such as computer-aided devices and the use of barcoded and Radio Frequency Identification labeled instruments, along with the implementation of fluorescence-coated needles, hold the potential to substantially diminish the RSIs and mitigate human error to a significant extent.
